# Compact Ultra-Wideband Monopole Antenna Loaded with Metamaterial

**DOI:** 10.3390/s20030796

**Published:** 2020-01-31

**Authors:** Samir Salem Al-Bawri, Hui Hwang Goh, Md Shabiul Islam, Hin Yong Wong, Mohd Faizal Jamlos, Adam Narbudowicz, Muzammil Jusoh, Thennarasan Sabapathy, Rizwan Khan, Mohammad Tariqul Islam

**Affiliations:** 1 Faculty of Engineering, Multimedia University, Persiaran Multimedia, Cyberjaya 63100, Selangor, Malaysia; shabiul.islam@mmu.edu.my (M.S.I.); hywong@mmu.edu.my (H.Y.W.); 2 Department of Electronics & Communication Engineering, Faculty of Engineering & Petroleum, Hadhramout University, Al-Mukalla 50512, Hadhramout, Yemen; 3 School of Electrical Engineering, Guangxi University, Nanning 530004, China; 4 Faculty of Mechanical Engineering, Universiti Malaysia Pahang, Pekan 26600, Pahang, Malaysia; faizaljamlos@ump.edu.my; 5 Department of Telecommunications and Teleinformatics, Wroclaw University of Science and Technology, 50-370 Wroclaw, Poland; adam.narbudowicz@pwr.edu.pl; 6 Bioelectromagnetics Research Group (BioEM), School of Computer and Communication Engineering, Universiti Malaysia Perlis (UniMAP), Kampus Pauh Putra, Arau 02600, Perlis, Malaysia; muzammil@unimap.edu.my (M.J.); thennarasan@unimap.edu.my (T.S.); 7 Department of Research and Development, Laird Technologies (M) Sdn Bhd, Penang 13600, Malaysia; rizwan.khan@lairdconnect.com; 8 Department of Electrical, Electronic and Systems Engineering, Faculty of Engineering and Built Environment, Universiti Kebangsaan Malaysia, UKM, Bangi 43600, Selangor, Malaysia

**Keywords:** monopole antenna, ultra-wideband (UWB), wideband, ENG metamaterial, near-zero refractive index (NZRI)

## Abstract

A printed compact monopole antenna based on a single negative (SNG) metamaterial is proposed for ultra-wideband (UWB) applications. A low-profile, key-shaped structure forms the radiating monopole and is loaded with metamaterial unit cells with negative permittivity and more than 1.5 GHz bandwidth of near-zero refractive index (NZRI) property. The antenna offers a wide bandwidth from 3.08 to 14.1 GHz and an average gain of 4.54 dBi, with a peak gain of 6.12 dBi; this is in contrast to the poor performance when metamaterial is not used. Moreover, the maximum obtained radiation efficiency is 97%. A reasonable agreement between simulation and experiments is realized, demonstrating that the proposed antenna can operate over a wide bandwidth with symmetric split-ring resonator (SSRR) metamaterial structures and compact size of 14.5 × 22 mm^2^ (0.148 λ_0_ × 0.226 λ_0_) with respect to the lowest operating frequency.

## 1. Introduction

In the past decades, an essential amount of research has been dedicated to planar antennas that allow one to use the increased spectrum demanded for modern wireless communication systems [1,2]. Nowadays, the development of planar wideband antennas supports applications requiring high data rates, very precise localization, and high-resolution radar systems [3,4,5]. One of many examples is Wireless Personal Area Networks (WPAN), where wide bandwidth antennas are required to cover both on-body and off-body communication [6]. The required antennas should offer small size, low cost, and be lightweight and able to be easily integrated with other circuitry. However, a narrow bandwidth is one of the drawback’s challenges that limits the usages of wideband modern wireless applications. To circumvent these challenges, various techniques have been developed recently. For instance, bandwidth and the radiation characteristics of an antenna can be improved using a reactive impedance surface (RIS) method [7]. Basically, by tuning RIS between magnetic and electric conductor (PEC and PMC) surfaces, antenna size can be miniaturized with a noticeable enhancement in bandwidth property. In reference [8], a significant impact on the antenna performance enhancement is demonstrated by applying a planar left-handed metamaterial (LHM) patterned structure on the bottom ground and upper patch of the dielectric substrate. This technique features capacitive-inductive characteristics due to the coupled bottom plane and upper patch configuration that can generate a traveling backward wave along the patch plane. However, a passive antenna with periodic structures on the bottom layers is applied and tested for temperature sensing, as presented in reference [9]. The bottom surface allows for significant enhancement in the bandwidth despite the compact antenna size.

Metamaterial was demonstrated to increase antennas’ bandwidth due to the ability to produce a negative refractive index (NRI) within both negative permeability (µ) and permittivity (ε) [10]. Furthermore, applying the metamaterial into the antenna design can offer increased gain, and good radiation patterns can be obtained [11]. However, a metamaterial structure with near-zero refractive (NZRI) properties has been investigated for specific applications, including several bands such as S, C, and X bands, to improve the antenna performance [12]. 

Monopole antennas are often preferred, as they offer small size, good radiation patterns, low cost, and high gain [13]; however, conventional antennas still suffer many challenges for both large size and narrow bandwidth. Therefore, advancements in metamaterial designs promise to mitigate those problems. Several types of metamaterial structures are proposed in the literature: split-ring resonators (SRRs) [14], complementary SRR (CSRR) [15], planar pattern, and capacitance-loaded strip (CLS) [16,17]. 

A compact UWB antenna is presented in reference [18] by appling multibranch T-shaped stub, which features a reconfigurable band notch and achieving a wide impedance bandwidth from 3 to 11 GHz. The aforementioned antenna is complicated. Therefore, a simplified UWB antenna design using a discrete embedded dielectric resonator (DR) structure has been proposed in [19] or semi-circular monopole antenna in [20]. The reported antennas obtained bandwidths form 1.44–18.8 GHz and 0.95–13.8 GHz, respectively. Vivaldi antenna in reference [21] is loaded with anisotropic zero-index metamaterial (AZIM) to realize a wideband frequency range of 1–10 GHz. Its overall size is 105 × 125 mm^2^, which is greater than the proposed antenna. In reference [22], a tapered slot antenna has been designed as well as several parallel-line unit cells with a broadband gradient refractive index (GRIN). An array of cells was placed at the forefront of the antenna to enhance the directivity, with antenna’s bandwidth being 3–11 GHz, which is less than the proposed work.

This paper presents a compact ultra-wideband monopole antenna using SNG metamaterial cells for wide bandwidth applications. The proposed key-shaped monopole with integrated SNG metamaterial cells offers a wide bandwidth from 3.08 GHz to 14.1 GHz. The metamaterial cells are located on the front and back sides of the antenna’s substrate. However, for the case of the antenna without SNG, the continuity of the bandwidth is disturbed, e.g., with a deteriorated impedance match for 11.5–14.1 GHz. Besides enabling wide bandwidth operation, SNG material allows a compact antenna size of 14.5 × 22 mm^2^ (0.148 λ_0_ × 0.226) with high gain, a quasiomnidirectional radiation pattern, and radiation efficiency ranging from 80% to 95%. To validate the efficacious of the proposed antenna, an overview of similar antennas published in the literature is reported in Table 1.

## 2. Unit Cell Design Architecture 

**Figure 1:**
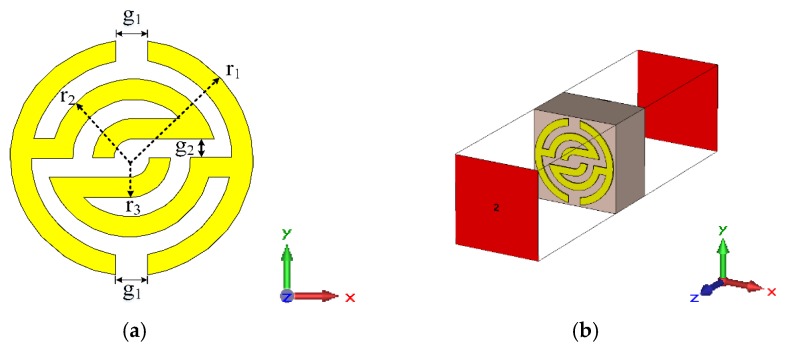
Metamaterial unit cell: (**a**) 2D view unit cell structure and (**b**) 3D view simulation setup (unit cell size 4.9 × 4.9 × 1.6 mm).

Figure 1a shows the proposed unit cell structure. It consists of several pairs of symmetric c-shaped split-ring resonators, which cover each other. The unit cell is designed by applying FR-4 substrate with 4.7 dielectric constant, and a thickness (*d*) of 1.6 mm. However, the conductor thickness is 0.035 mm. Finite integration technique (FIT)-based CST Microwave Studio computer simulation software has been used to design and simulate the unit cell. The proposed structure is simulated over 0.1–15 GHz by placing the structure between two waveguide ports situated on each side of the z-axis that was used for the electromagnetic excitation as shown in Figure 1b. However, perfect electric conductor (PEC) and perfect magnetic conductor (PMC) are deployed as boundary conditions along the x-axis and y-axis, respectively. The dimensions specifications of the proposed unit cells are illustrated in Table 2. 

**Figure 2:**
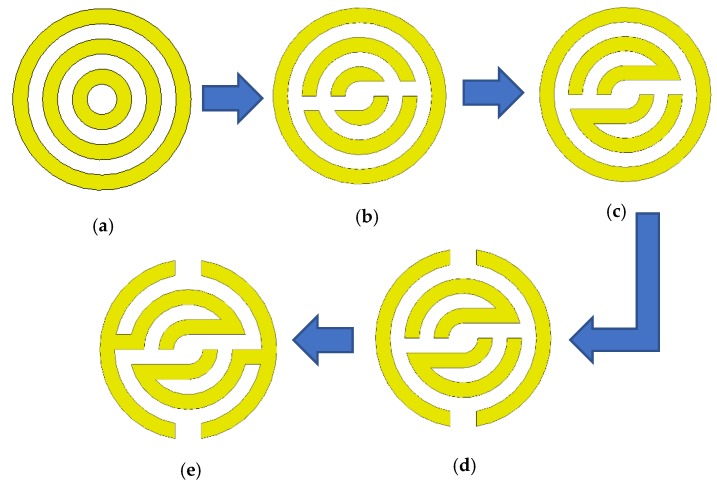
Schematic views for the evaluation of the proposed MTM.

The proposed metamaterial (MTM) unit cell is composed of several metallic rings and separated by 0.5 mm gape (see Figure 2a). Each inner ring has two splits; however, they are connected together using a metal strip with a 0.5 mm width as shown in Figure 2b,c, respectively. The outer ring is composed of two narrow gapes, and they are spaced 180 degrees apart from each other, as demonstrated in Figure 2d. Furthermore, the stopband increases in the case of using two thin arms between the inner and outer rings (see Figure 2e), which is the final optimize MTM unit cell. The metal rings act as inductors, while the gaps between the outer and inner rings act as capacitors. Figure 2 summarizes the evaluating steps of the proposed MTM. 

## 3. Metamaterial Unit Cell Working Principle

**Figure 3:**
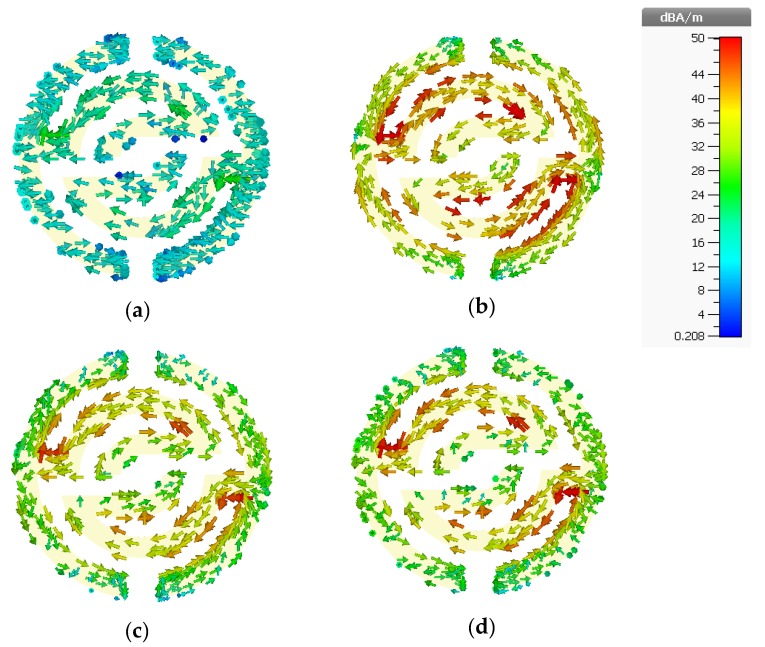
Surface current distribution at (**a**) 3.5 GHz, (**b**) 10.3 GHz, (**c**) 11 GHz, and (**d**) 11.9 GHz.

To observe the proposed metamaterial behavior and understand the physical phenomena of how it works when it is located into an electric and magnetic field region, the surface current distributions are analyzed and discussed for different frequencies. The surface current distributions of the proposed MTM unit cell at 3.5 GHz, 10.3 GHz, 11 GHz, and 11.9 GHz are illustrated in Figure 3a–d. The arrows indicate the current distribution direction in the overall structure, and the colors represent the current intensity. In Figure 3a, weak distributed surface current can be observed clearly via the overall structure at 3.5 GHz. However, strong surface current at 10.3 GHz is present across the whole structure, especially at edges and corners of the inner symmetric C-shaped structure, as shown in Figure 3b, although the current distribution flows in opposite directions regarding the upper and down C-shaped etching strips, which are nullifying the current and generates a stop band for frequencies above 9.8 GHz. 

In Figure 3c,d, the surface current distribution behavior is relatively in fluctuation mode at the symmetrical C-shape of outer and middle ring’s regions, whereas currents flow in two different directions. Furthermore, those two anti-symmetric conductor currents are observed at the resonance, which can be depicted as an equivalent magnetic moment, whereas the featured artificial magnetism of the proposed structure is generated in this magnetic moment, which can cause the influential negative permeability of the metamaterial structure [25]. 

**Figure 4:**
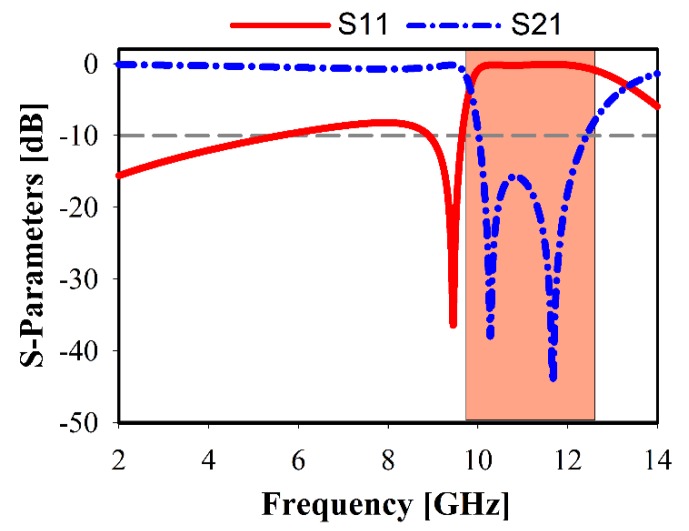
Simulated metamaterial reflection and transmission coefficients.

The simulated reflection coefficient (*S*_11_) and the transmission coefficient (*S*_21_) results of the cell are demonstrated in Figure 4. It shows a stop-band in the range of 9.8–12.8 GHz, which is applicable for some applications, which are operating within this range like the Ku band (downlink) and the highest frequencies of UWB. The outer and inner split ring-shaped resonators are considered as the sufficient cause to achieve an extensive stopband operation, which has a higher resonance at 11.9 GHz (denoted by a blue dotted line). 

**Figure 5:**
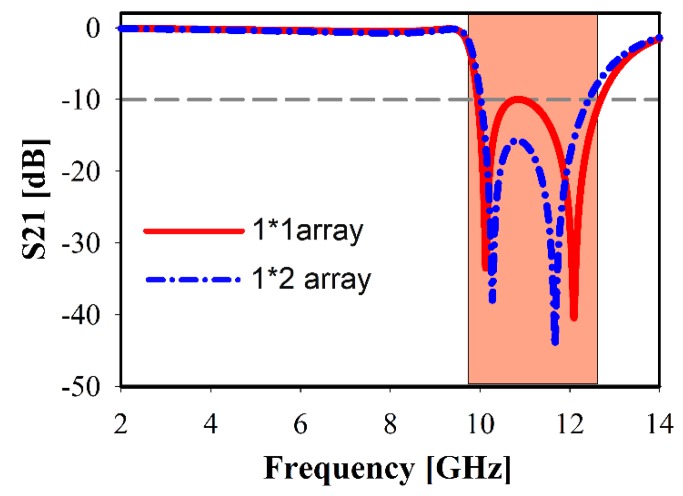
Transmission coefficients for different unit cell array structures.

Metamaterial structure conducts like an inductance–capacitance (LC) resonant circuit. Two characteristics properties are considered for creating the metamaterial resonances: firstly, a split gap and then a metal strip. The metal strip line is excited via the magnetic field, which is parallel to the metal axis. However, the electric field is caused in the unit cell split gape. The LC resonance frequency leads to a decrease or increase in the resonance towards lower or higher frequency by controlling the split gap capacitance [25]. Here, the selected gap is considered as a capacitor, while the C-shaped resonators, including the rectangular small copper arms, act as an inductor that can control the metamaterial resonant characteristics. Figure 5 shows the transmission coefficients of different unit cell arrays of 1 × 1 and 2 × 1 array structures. The 1 × 1 array structure indicates resonance at the range of 9.8–12.8 GHz whereas better match exhibits for 2 × 1 array. 

Robust method is implemented to characterize the effective parameters from the normal incidences scattering parameters data [26,27]. It starts by presenting the transmission (*S*_21_) and refraction coefficients (*S*_11_) compound terms, whereas the simulations are performed in the frequency range between 2 and 14 GHz. Based on that method, Equations (1) and (7) are used to retrieve the effective parameters.

(1) $$S_{11}=\;\left(\frac{R_{01}\;\left(1-\;e^{i2nk_{0}d}\right)}{1-\;R_{01}{}^{2}\;e^{i2nk_{0}d}}\right)$$

(2) $$S_{21}=\;\left(\frac{\;\left(1-\;R_{01}{}^{2}\;\right)\;e^{ink_{0}d}}{1-\;R_{01}{}^{2}\;e^{i2nk_{0}d}}\right)$$

where $k_{0}$ is the wave vector in free space and d is the prototype thickness and R01= z−1 z+1.

According to the field theory, dielectric-metal interface (metamaterial) is considered as a passive medium in electromagnetic filed circumstances. Therefore, impedance imaginary part and refractive index real part depend on Equations (3) and (4).

(3) real (z) ≥0

(4) imaginary (n) ≥0

Based on that condition, impedance can be calculated by the Equation (5).

(5) $$z=\;\pm \;\sqrt{\frac{{\left(1+\;s_{11}\right)}^{2}-\;s_{21}{}^{2}}{{\left(1-\;s_{11}\right)}^{2}-\;s_{21}{}^{2}}}$$

(6) $$e^{ink_{0}d}=X\;\pm i\sqrt{1-\;X^{2}}$$

where X= 12 s21 (1− s112+ s212 ) .

The refractive index can be calculated by the Equation (7), where the real part of the refractive index n is the branch of the logarithm function.

(7) $$n=\;\frac{1}{k_{0}d}\;\left\{\left[imaginary\;\left[\ln e^{ink_{0}d}\right]+2m\pi \right]-i\;\left[real\;\left[\ln e^{ink_{0}d}\right]\right]\right\}$$

where *m* is the integer value. 

**Figure 6:**
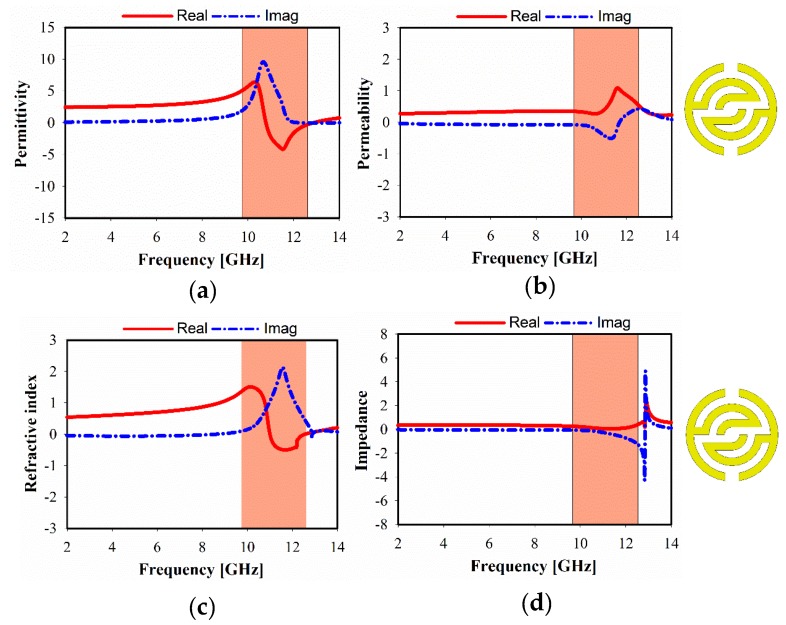
Metamaterial, simulated results of 1 × 1 unit cell: (**a**) permittivity, (**b**) permeability, (**c**) refractive index, and (**d**) impedance.

**Figure 7:**
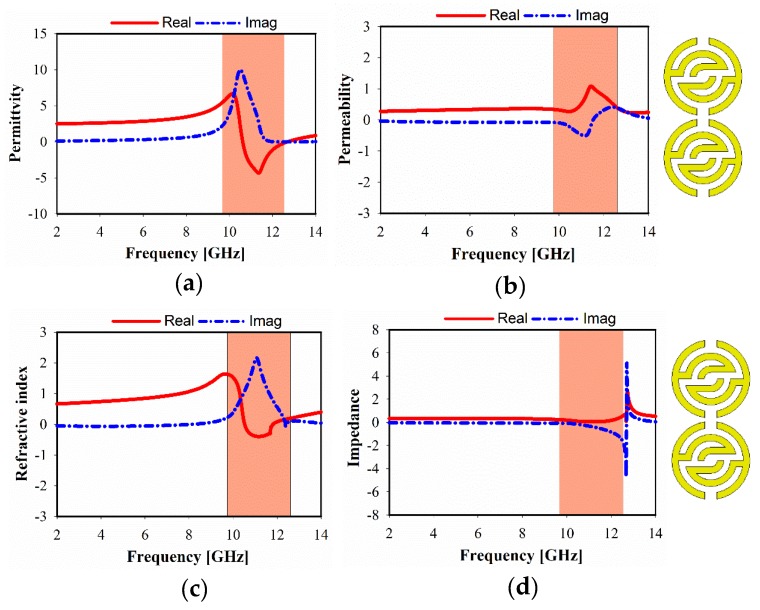
Metamaterial, simulated results of 2 × 1 unit cell: (**a**) permittivity, (**b**) permeability, (**c**) refractive index, and (**d**) impedance.

**Figure 8:**
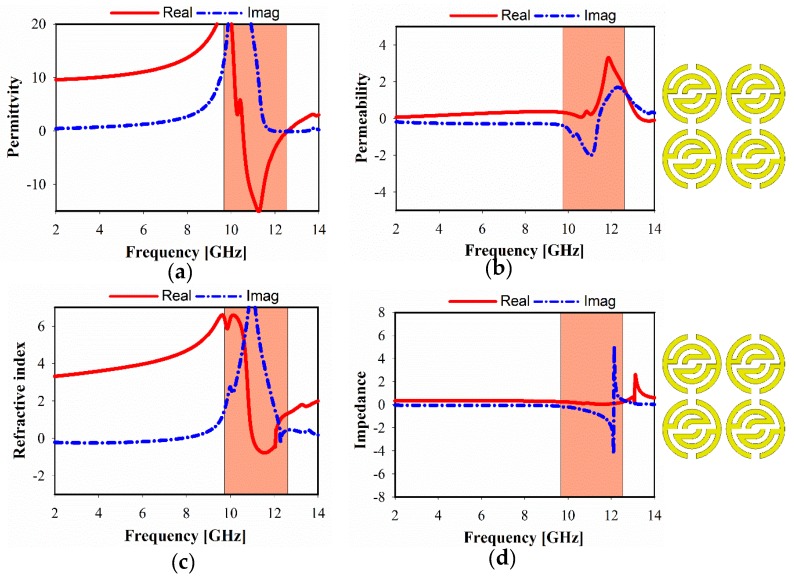
Metamaterial, simulated results of 2 × 2 unit cells: (**a**) permittivity, (**b**) permeability, (**c**) refractive index, and (**d**) impedance.

The simulated relative real and imaginary parts of the effective permittivity, permeability, refractive index, and impedance, of different unit cell array structures are plotted in Figure 6, Figure 7 and Figure 8. For 1 × 1 unit cell array, as shown in Figure 6, a real negative permittivity (ε) value exhibits for the frequency range from 10.7 to 12.85 GHz. However, it is remarkable that a greater than 1.5 GHz bandwidth can be achieved with a near-zero refractive index (NZRI) property in z-axis wave propagation. In Figure 7, it is apparent that a different number of unit cells exhibit a slight variation in the characteristics of metamaterial over the frequency range. As a result, SSRR loading with 2 × 1 unit cell arrays provides single epsilon-negative (ENG) characteristics over the band (10.6 to 12.7 GHz), as demonstrated in Figure 7a, as well as near-zero refractive index (NZRI) value over the range from 10.5 to 11.8 GHz. Figure 8 shows the simulated characteristics of 2 × 2 MTM array structure. It is obviously seen that the near-zero refractive index (NZRI) has been exhibited over the range of 10.8–12.1 GHz. However, 10.4–12.5 GHz is the effective real value range of the displayed ENG, whereas permeability shows Mu (µ) near-zero (MNZ) in the frequency range of 9.6–11.2 GHz at z-axis wave propagation.

## 4. Configuration of the Proposed Antenna

**Figure 9:**
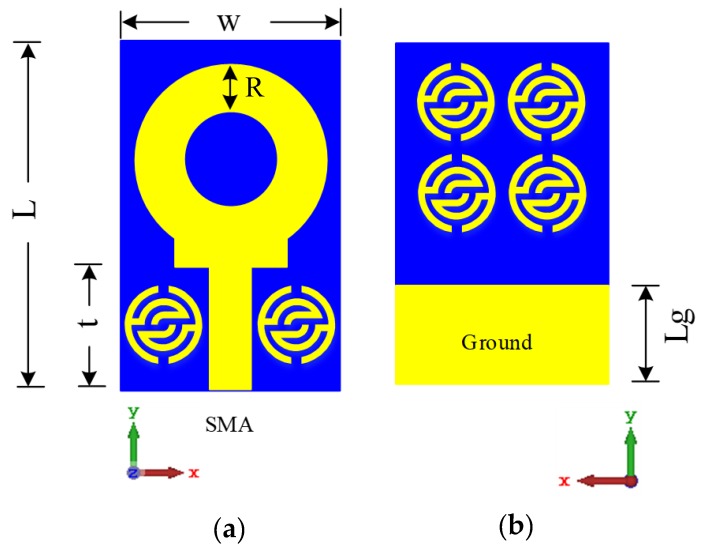
Geometry of the proposed antenna: (**a**) front view and (**b**) back view.

Figure 9a,b illustrate the proposed antenna structure designed on the FR-4 substrate of 1.6 mm thickness with a loss tangent of 0.025 and relative permittivity of 4.7. The overall size including the substrate is 22.5 × 14 × 1.6 mm^3^. A 50-Ω SMA connector is used to feed the antenna. The parameters of the proposed antenna and unit cell dimensions are listed in Table 2. It can be seen that 2 × 2 unit cells are placed on the antenna at the reverse side above the partial ground plane, whereas two unit cells are placed horizontally side-by-side to the antenna’s key-shaped monopole features refractive index. 

## 5. Results and Discussion

**Figure 10:**
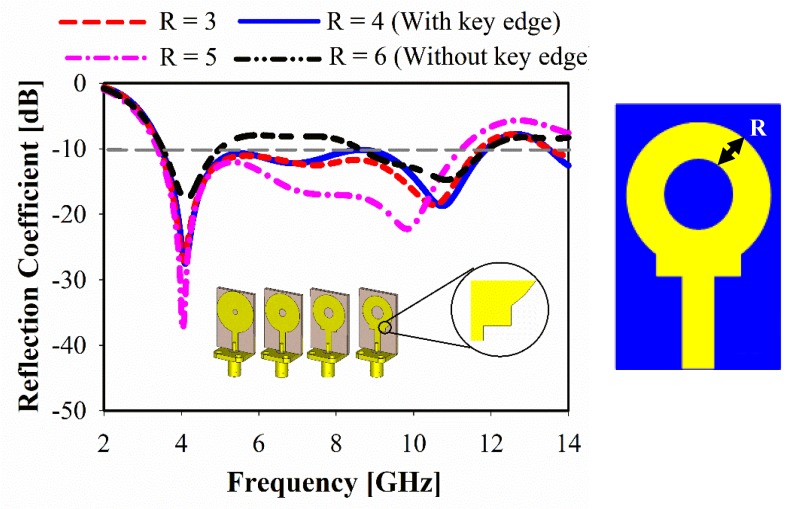
Simulated reflection coefficient for ring radiator line-width R.

The effect of various ring radiator line-width (*R*) to the proposed antenna performance is shown in Figure 10. Impedance mismatch was found on some frequency ranges (4.9–8.7 GHz and 11.5–14.1 GHz) for *R* = 6 mm. It can be observed that those bands are shifted slightly towards the upper frequency, where the lower band is matched at 3 mm optimum value of *R*. Additionally, an additional spike at the base of the monopole is added to ensure good impedance match, as shown in Figure 10. 

**Figure 11:**
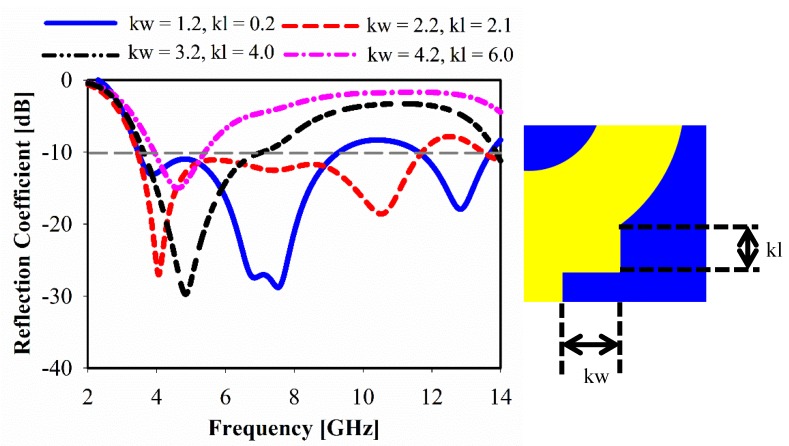
Simulated reflection coefficient for different width and length of key edge.

Both width (*kw*) and length (*kl*) of the key edge are composed of 1 mm and 2 mm increments, respectively. Changing of the key edge structure effectively shifts the resonance towards lower frequencies, as shown in Figure 11. When the *kw* and *kl* are kept larger, narrow bandwidth can be observed, whereas the resonance at high frequency starts to disappear. The final optimized dimensions of the key edge for each side are *kw* = 2.2 and *kl* = 2.1.

**Figure 12:**
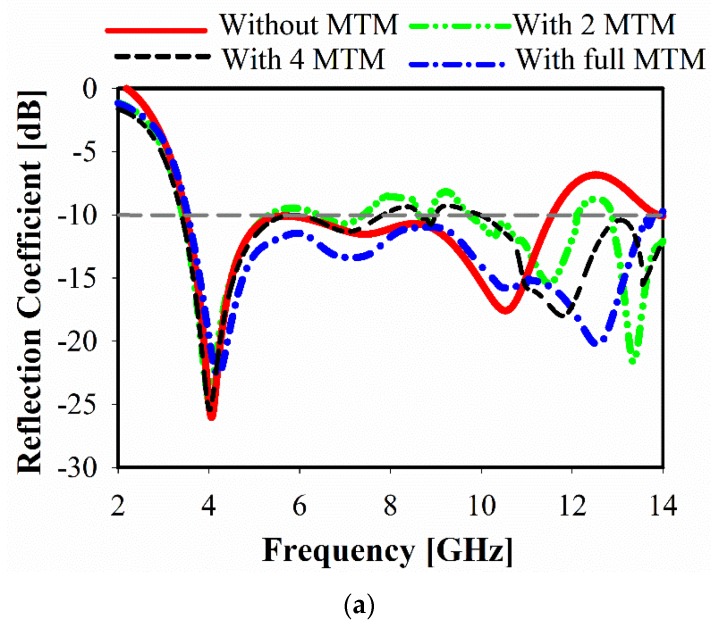

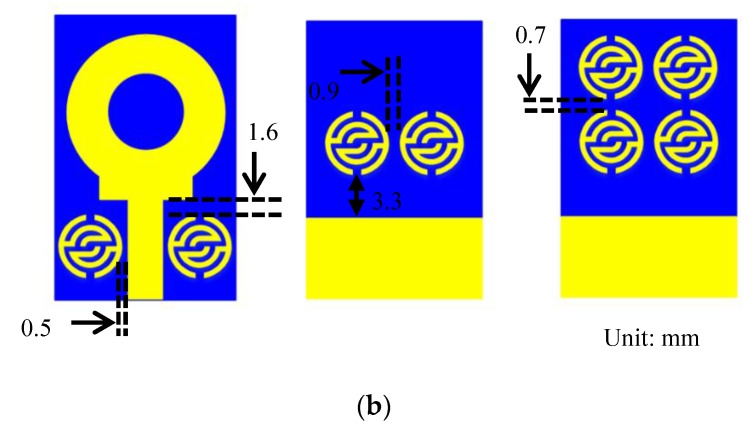
Simulated reflection coefficient with and without MTM. (**a**) the reflection coefficient of the proposed antenna without and with MTM; (**b**) the investigation of efficient positions of proposed MTM with the antenna.

The antenna resonant frequency can be adjusted by changing the place and number of MTM unit cells. In order to observe the influence of the MTM unit cells on antenna performance, the reflection coefficient of the proposed antenna without and with MTM is discussed, as shown in Figure 12a; whereas the investigation of efficient positions of proposed MTM with the antenna is revealed in Figure 12b. In the case of no MTM applied, it can be seen that impedance mismatching has occurred at high frequency. However, the resonant mode at 10.5–14 GHz will be excited by applying only two MTM unit cells on the antenna bake side (close to the partial ground). Furthermore, good impedance matching and wider bandwidth are gained with the use of four MTM unit cells, including two near to the transmission line.

**Figure 13:**
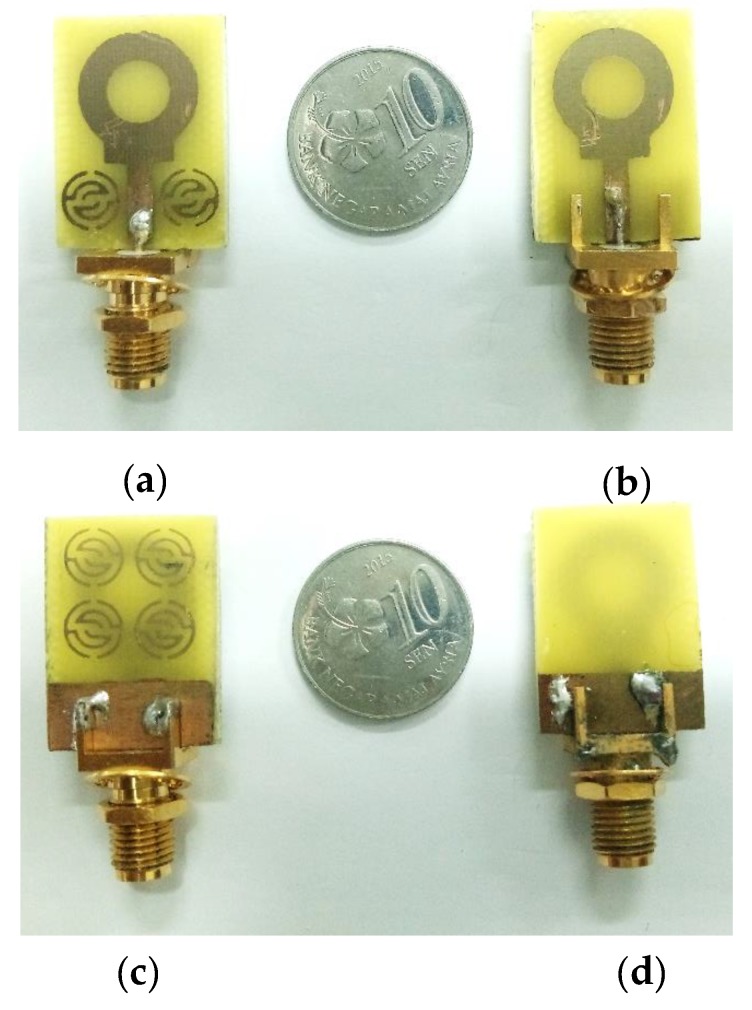
Fabricated prototypes of the proposed antennas, front view: (**a**) with MTM and (**b**) without MTM; back view: (**c**) with MTM and (**d**) without MTM.

**Figure 14:**
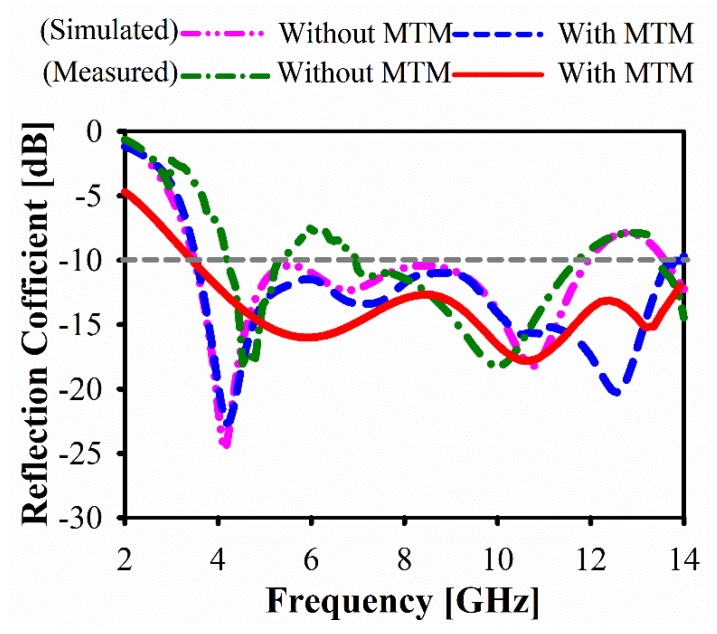
Simulated and measured reflection coefficient (*S*_11_).

Two antennas have been fabricated with and without metamaterial, as shown in Figure 13. The reflection coefficients (*S*_11_) of these prototypes are measured using an Agilent E8051C Network Analyzer (ENA) and compared with simulation results, as demonstrated in Figure 14. Regarding the reflection coefficients (*S*_11_), the measured bandwidths for both cases are similar, stretching from 3.08 GHz to 14.1 GHz, which fulfills ultra-wideband requirements, as shown in Figure 14. However, for the antenna without MTM, small peaks are seen at higher frequencies that exceed the −10 dB limits, preventing wideband performance. The introduction of MTM allowed for the suppression of those peaks, providing continuous operation within the whole bandwidth.

**Figure 15:**
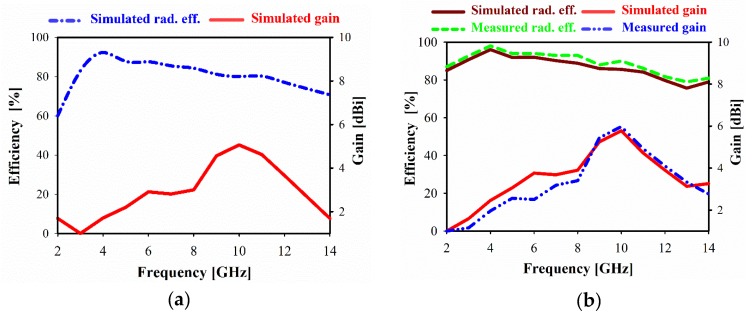
The proposed antenna gains and radiation efficiency over frequency (**a**) without MTM and (**b**) with MTM.

Figure 15 illustrates the simulation and measurement of both radiation efficiency (denoted as rad. eff.) and gains. It can be seen that the realized gain is close to omnidirectional antenna at lower frequencies and gradually increases with frequency up to 6.12 dBi due to larger antenna’s aperture compared to the wavelength. The achieved gain decreases slightly at higher frequency range due to the insertion loss caused by using FR-4 substrate and degradation rate of the distributed power on those bands. In the fabricated PCB circuits using FR-4, the significant loss is observed at higher frequencies due to the increase of its dielectric constant, which has a higher dissipation factor (Df) and feed line radiation. However, decrement in the measured result was obtained due to the fabrication precision issues of the very compact size of the unit cells. Besides, the simulated radiation efficiency in the whole range varies between 80% and 97%. A good agreement between the simulated and measured results was observed. 

**Figure 16:**
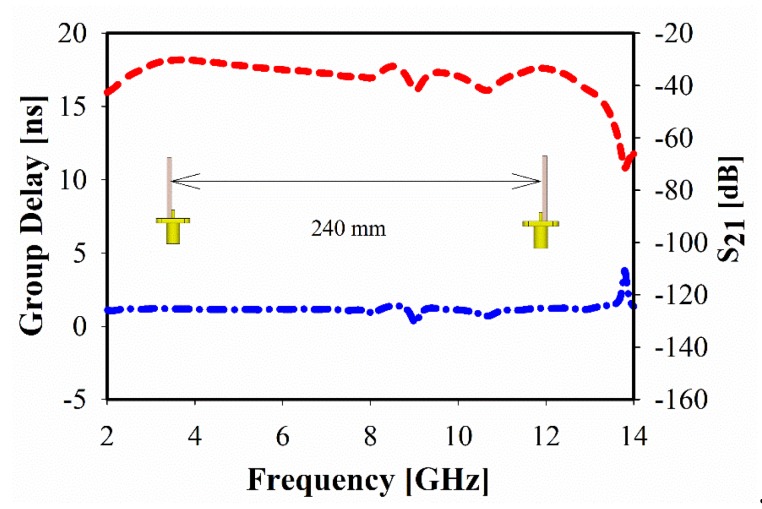
Group delay response of face-to-face position.

The simulated face to face group delay (GD) of the proposed UWB antenna is shown in Figure 16. It is almost uniform with less variation (<1.3 ns) over the UWB frequency range. As a result, it can be seen that the signal between the transmitting and the receiving UWB antennas system was not distorted. Moreover, the *S_21_* transfer function (TF) in Figure 16 shows less distortion, except at higher bands above 13 GHz. Accordingly, the realized TF, as well as the flat GD over the UWB frequency range, makes this antenna suitable to be used for short-range communications applications.

**Figure 17:**
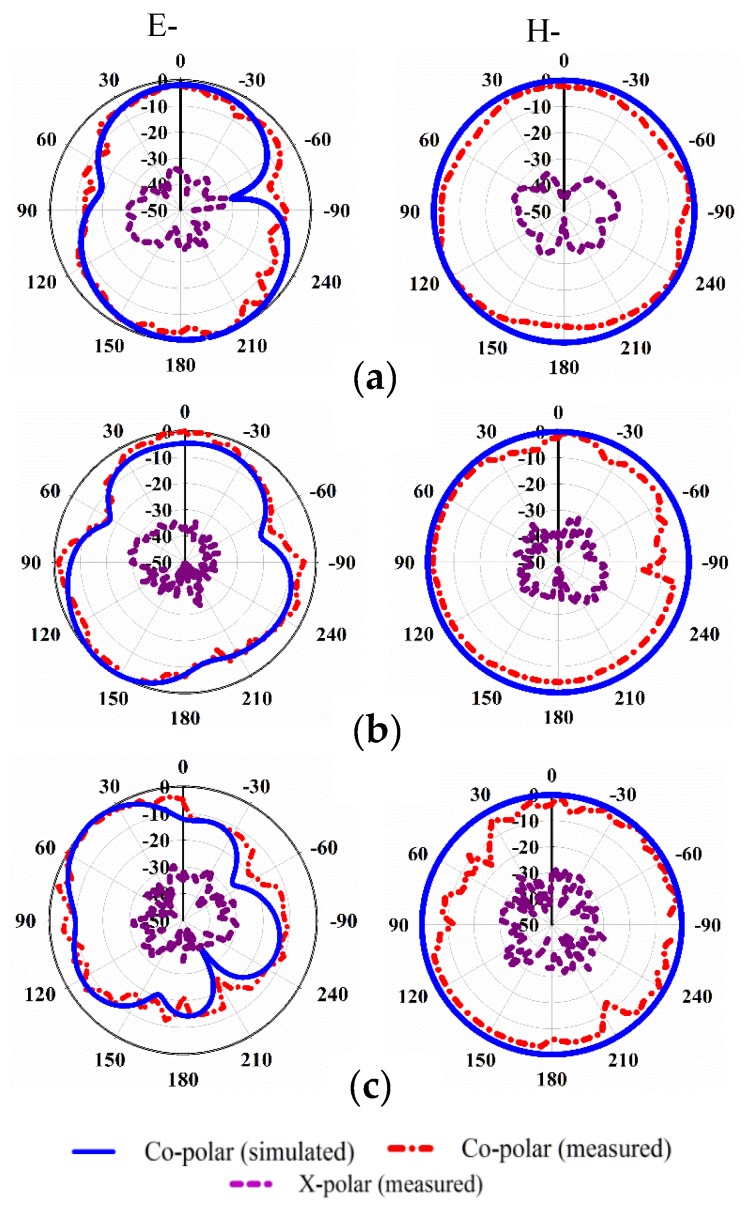
Simulated and measured radiation patterns of the proposed antenna at: (**a**) 4 GHz, (**b**) 8 GHz, and (**c**) 11 GHz.

However, the normalized radiation patterns in H-plane and E-plane are shown in Figure 17 for 4 GHz, 8 GHz, and 11 GHz. A bidirectional behavior can be seen at 4 and 8 GHz, while nearly uni-directional pattern occurs at the higher frequencies of 11 GHz, which is affected by the back MTM. Furthermore, losses have been occurred at higher frequencies due to the difficulties in the fabrication process of the compact metamaterial antenna. Meanwhile, a slight degradation might be found in both radiation and total efficiencies [28,29].

## 6. Conclusions

A compact ultra-wideband (UWB) antenna integrated with an array of metamaterial cells with SNG and NZRI characteristics is presented in this paper. A unique symmetric split-ring resonator (SSRR) metamaterial unit cell is proposed. It features a wide bandwidth with a negative refractive index (NRI), spanning from 9.67 GHz to 12.11 GHz. The proposed unit cell exhibits SNG features in different bands. Several unit cells are then placed on the backside of the antenna and close to the antenna’s transmission line. The simulated reflection coefficient (*S*_11_) shows a wide bandwidth of 128.3% (from 3.08 to 14.1 GHz). 

## Figures and Tables

**Table 1:** Comparison of the designed antenna with others in the state of art.

Ref.	Size W × L (mm^2^)	Operating Freq. Rang (GHz)	Technique	10-dB BW (%)	Max. Gain (dBi)
[6]	27.00 × 33.00	3.20–14.00	Metamaterial	126	3.90
[18]	40.00 × 20.00	3.00–11.00	T-shaped ground	114	5.00
[19]	17.60 × 33.60	3.20–10.96	- - -	110	3.30
[23]	12.00 × 22.00	3.10–11.10	Slots	114	4.00
[24]	40.00 × 40.00	3.10–11.00	Vivaldi antenna	114	7.06
This work	14.50 × 22.00	3.08–14.10	NZRI/SNG metamaterial	128.3	6.12

**Table 2:** Parameters dimensions of the proposed unit cell and antenna.

Para.	Value (mm)	Para.	Value (mm)	Para.	Value (mm)	Para.	Value (mm)	Para.	Value (mm)
g1	0.7	r1	3	r3	1	L	22.5	t	8
g2	0.5	r2	2	R	3	W	14	Lg	6.5
